# Long Non-Coding RNA TUG1 Gene Polymorphism and TUG1 Expression Level as Molecular Biomarkers of Systemic Lupus Erythematosus and Lupus Nephritis

**DOI:** 10.3390/ncrna9050056

**Published:** 2023-09-19

**Authors:** Gehan Abd-Elfatah Tawfeek, Heba Kasem, Eman Ali Abdallah, Mohammed Almulhim, Abdullah Almulhim, Mohammed Albarqi, Khaled Mohamed Amin Elzorkany

**Affiliations:** 1 Clinical Pathology Department, Faculty of Medicine, Menoufia University, Shebeen El-Kom 32511, Egypt; 2 Nephrology Unit, Internal Medicine Department, Faculty of Medicine, Menoufia University, Shebeen El-Kom 32511, Egyptkhaledelzorkany@med.menofia.edu.eg; 3 Rheumatology, Rehabilitation and Physical Medicine Department, Faculty of Medicine, Menoufia University, Shebeen El-Kom 32511, Egypt; 4 Internal Medicine Department, College of Medicine, King Faisal University, Al-Ahsa 31982, Saudi Arabia; 5 Family and Community Medicine Department, College of Medicine, King Faisal University, Al-Ahsa 31982, Saudi Arabia

**Keywords:** systemic lupus erythematosus, lupus nephritis, lncRNA TUG1 SNPs, TUG1 expression

## Abstract

Long non-coding RNA (lncRNA) TUG1 acts as a proto-oncogene, allowing the proliferation of tumor cells, and it has been related to inflammation. Therefore, we aimed in this study to investigate for the first time the role of TUG1 gene polymorphism and the TUG1 level as biomarkers in systemic lupus erythematosus (SLE) and their link to lupus nephritis 145 SLE. A total of 145 healthy controls were subjected to clinical and laboratory evaluation. The disease activity was assessed by the SLE disease activity index (SLEDAI) score. SLE patients were divided into two subgroups according to the presence of lupus nephritis. The TUG1 gene polymorphisms rs5749201 and rs886471 were determined by Sanger sequencing, and TUG1 expression was assessed by qRT-PCR. There was a significant increase in the risk of SLE AA, TA, dominant genotypes, and the A allele of rs5749201 (*p* < 0.001) by 4.9-, 10.1-, 6.5-, and 2.5-fold in comparison to the relative control. GG and TG, dominant genotypes and the G allele of rs886471 (*p* < 0.01) increased the risk by 5.09-, 11.9-, 6.5-, and 2.6-fold. AA, A allele, dominant and recessive rs5749201genotypes increased the risk of lupus nephritis by 16.6-, 7.4-, 7.1-, and 12.2-fold, respectively (*p* < 0.05). GG, dominant and recessive genotypes, and the G allele of rs886471 increased the risk of lupus nephritis by 17.04-, 7.8-, 9.4-, and 6.08-fold, respectively (*p* < 0.05). Additionally, the AG haplotype increased the risk of SLE and lupus nephritis by 2.7- and 7.8-fold, respectively. The AA rs5749201 and GG rs886471 variants are significantly associated with more severe disease (*p* < 0.001). TUG1 expression was significantly higher in SLE than in the control and in the lupus nephritis than in non-lupus nephritis cases (*p* < 0.05). Interestingly, AA rs5749201 and GG rs886471 were significantly associated with higher TUG1 levels (*p* < 0.001). It was also found that AA rs5749201 and high SLEDAI were predictors of lupus nephritis. Overall, our findings illustrated for the first time that TUG1 gene rs5749201 and rs886471 variants were associated with increased risk of SLE, more severe disease, and lupus nephritis, and the TUG1 level could be used as a diagnostic biomarker of SLE and lupus nephritis.

## 1. Introduction

Systemic lupus erythematosus (SLE) is a chronic autoimmune disease with a wide variety of manifestations ranging from mild cutaneous to organ failure as lupus nephritis (LN) and cardiopulmonary complications [1]. SLE is mainly present among young women, with a greater incidence in certain ethnic groups, such as Asian, Black, and Hispanic populations. SLE increases the mortality rate in young women to 2.6 times more than that of the general population [2].

A defect in self-tolerance is considered the main mechanism in the development and complications of SLE because of the production of autoantibodies and the formation of immune complexes, which precipitate in tissues leading to chronic inflammation and organ damage [3]. Many other factors, including genetic, environmental, and hormonal, are proven to contribute to SLE; however, the pathogenesis is complex and still needs more research [4].

A better understanding of the pathogenesis of SLE can facilitate the identification of vulnerable patients, discovery of therapeutic objectives, and the design of prevention tests. Several genetic sites, such as the major histocompatibility complex, Fc-gamma receptors, the signal transmitter, and transcription activator gene, were associated with the risk of SLE in different populations. However, most genetic variations that have been recognized so far are estimated to have less than 10% of SLE genetic sensitivity [5].

The currently accessible diagnostic markers for lupus diagnosis are sub-optimal. The ANA test had high sensitivity (94%) but relatively low specificity (61%). On the other hand, anti-dsDNA and anti-SM antibodies have higher specificity to SLE, but lower sensitivity due to their transitory existence. To reach better outcomes, continuous assessment and surveillance of the disease progression and prediction of the lupus course are necessary. Thus, more precise and robust biomarkers are needed for monitoring disease progression, drug evaluation, and prediction of upcoming lupus flare [6].

Non-coding RNA is one of the most important factors in epigenetics, and its regulatory role in genetic research is currently an essential area [7]. Long non-coding RNA (lncRNA) has been investigated in many studies as an essential factor in many diseases, such as heart diseases [8], neurodegenerative diseases [9], immunological disorders [7], and cancer [10].

Although the functions of most lncRNAs are still under investigation, they have important roles in most subclasses of immune cells, such as regulating immune cell differentiation, homeostasis, and effector function [11]. Compared to mRNAs, lncRNAs are more expressed in a tissue- and disease-specific manner, making them attractive therapeutic targets and biomarkers for disease diagnosis [12,13]. In addition to the identification of specific lncRNAs in immune cells, which helps in understanding the function of the non-coding portions of the human genome, these may also be used as biomarkers for immunologic diseases and eventually may reveal novel therapeutic targets [7].

One lncRNA currently being studied is the Taurine-upregulated gene 1 (TUG1), 7.1 kb in length, located on chromosome 22q12.2. [5]. It was found that TUG-1 can suppress and silence the target genes, affecting their expression, and competing for transcription factors [5].

Recently, several functions of TUG1 have been explored. TUG1 is reported to be highly expressed in multiple cancers, such as bladder cancer, hepatocellular carcinoma, glioma, and osteosarcoma [14,15].

Previous studies have proven that TUG1 acts as a proto-oncogene, allowing for the proliferation of tumor cells [16]. Moreover, it was found that it affects the process of insulin secretion and apoptosis in pancreatic tissues. The inflammatory response reported that a TUG1 knockdown occurred, associated with a decrease in hyperlipidemia and a decrease in the release of inflammatory cytokines, and lncRNA TUG1 was upregulated in LPS-induced hepatocyte inflammation by targeting miR140-TNF [17,18]. It is also associated with the risk of atherosclerosis through modulating the miR-21/phosphatase and tensin homolog (PTEN) axis [19]. Therefore, it has been related to inflammation and we hypothesize that TUG1 may be related to SLE. To the best of our knowledge, no previous studies have investigated the TUG1 gene SLE. The current study investigated the lncRNA TUG1 levels and TUG1 rs5749201 and rs886471 single nucleotide polymorphisms (SNPs) as risk and biomarkers of SLE and LN and relation to clinical laboratory parameters.

## 2. Results

### 2.1. Characteristics of the Study Subjects

In the present study, 145 SLE cases with 145 age and gender-matched healthy control were included; 85.5% were females, and 13.5% were males with a median age of 42.0 (19–53 years old) and median disease duration of 5 years (0.5–20); 48.3% of patients were diagnosed as LN (Table 1).

### 2.2. LncRNA TUG 1 Genotype Distributions, Alleles and Risk of SLE

Our results demonstrated that genotype distribution was in accordance with Hardy–Weinberg equilibrium in both cases (^HW^χ^2^ = 3.629, *p* = 0.057 *for* rs5749201 and ^HW^χ^2^ = 3.256, *p* = 0.071 *for* rs886471) and control (^HW^χ^2^ = 3.542, *p* = 0.06 *for* rs5749201 and ^HW^χ^2^ = 3.734, *p* = 0.053 *for* rs886471). There was significant difference between the control and SLE cases regarding the genotype distribution of TUG1 rs5749201 and rs886471 (*p* < 0.001), Table 2.

It was found that AA, TA, dominant genotypes, recessive and A allele of rs5749201 increased the risk of SLE (*p* < 0.001) by 12.1-fold (CI = 5.951–24.799), 3.3-fold (CI = 1.672–6.553), 6.1-fold (CI = 3.263–11.468), 5.6-fold (CI = 3.343–9.680 and 4.3- fold (CI = 3.041–6.136) respectively of the relative control. rs886471 GG, TG, dominant genotypes, recessive and G allele increased the SLE risk (*p* < 0.01) by 13.3-fold (CI = 6.557–27.27), 3.6-fold (CI = (1.862–7.06), 6.5-fold (CI = 3.545–12.1), 6.03-fold (CI = 3.545–12.105) and 4.5-fold (CI = 3.195–6.4) respectively of the relative control, Table 3.

### 2.3. LncRNA TUG 1 Genotype, Alleles and Risk of Lupus Nephritis

As regarding TT and AA are the reference genotypes of rs5749201 and rs886471 respectively, SLE cases with AA & A allele rs5749201 and GG &G allele rs886471 carry the risk of LN by 16.67-fold (C.I = 3.487–79.72, *p* < 0.001); 7.4-fold (C.I = 3.878–14.393, *p* < 0.001) and 17.04-fold (C.I = 3.585–81.03, *p* < 0.001); 6.0-fold (C.I = 3.301–11.234, *p* < 0.001). Dominant and recessive of both SNPs carry the risk of LN by 7.12-fold (C.I = 1.547–32.86, *p* = 0.012); 12.13-fold (C.I = 5.397–27.25, *p* < 0.001); 7.8-fold (C.I = 1.704–35.73, *p* = 0.008); 9.0-fold (C.I = 4.212–19.42, *p* < 0.001) respectively (Table 4).

### 2.4. Effect of Haplotypes on the Disease Risk

It was found that haplotypes AT and AG significantly increased the risk of SLE by 2.7-fold (*p* = 0.046) and 4.7-fold (*p* < 0.001), respectively relative to control. Additionally, AG haplotype increased the risk of LN by 7.8-fold (*p* < 0.001) relative to non-LN cases. However, none of the other haplotypes carry risks (Table 3 and Table 4).

### 2.5. Relation of rs5749201 and rs886471 SNPs of the TUG 1 with Clinic Pathological Features of SLE

To evaluate the effect of lncRNA TUG 1 gene polymorphism on clinical pathological features of SLE, clinic laboratory parameters among the different genotypes were evaluated, and there was a significant association of AA rs5749201 and GG rs886471 genotypes with higher age (*p* < 0.001; *p* < 0.001), higher male percentage (*p* = 0.019; *p* < 0.043), longer diseases duration (*p* = 0.001; *p* < 0.001), musculoskeletal affection (*p* < 0.001; *p* < 0.001), leucopenia (*p* < 0.001; *p* < 0.001), thrombocytopenia (*p* < 0.001; *p* < 0.001), higher CRP (*p* = 0.004; *p* < 0.001), ESR (*p* < 0.001; *p* < 0.001), C3 (*p* < 0.001; *p* < 0.001), and C4 (*p* < 0.001; *p* < 0.001), more percentage of positive double-stranded DNA (dsDNA) (*p* < 0.001; *p* < 0.001), higher SLEADI index (*p* < 0.001; *p* < 0.001), and more percentage renal affection (*p* < 0.001; *p* < 0.001). Additionally, the GG rs886471 genotypes were associated with lower Hb level (*p* = 0.016) but no significant correlation with other parameters (*p* > 0.05). In addition, heterozygote TA rs5749201 was associated with arthritis (*p* < 0.001), leucopenia (*p* < 0.001), thrombocytopenia (*p* = 0.004), higher C4 (*p* = 0.034), more dsDNA percentage (*p* < 0.001), and higher SLEDAI score (*p* = 0.003). TG rs886471 genotype was also associated with leucopenia (*p* = 0.001), thrombocytopenia (*p* = 0.004), more dsDNA percentage (*p* < 0.001), and higher SLEDAI score (*p* = 0.005) (Table 5 and Table 6).

### 2.6. LncRNA TUG 1 Level in the Studied Groups

**Figure 1:**
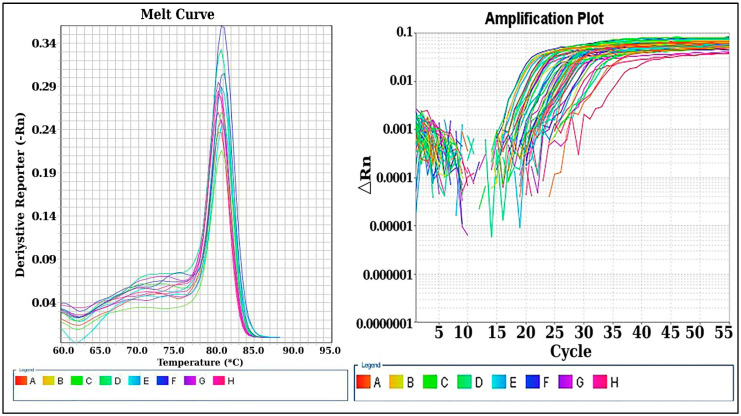
LncRNA TUG1 expression in cases and control by qRT-PCR. Ct values were processed using 2^−ΔΔCT^ method, and TUG1 expression was normalized to U6 endogenous control. Post-amplification melting-curve analysis ensured reaction specificity.

**Figure 2:**
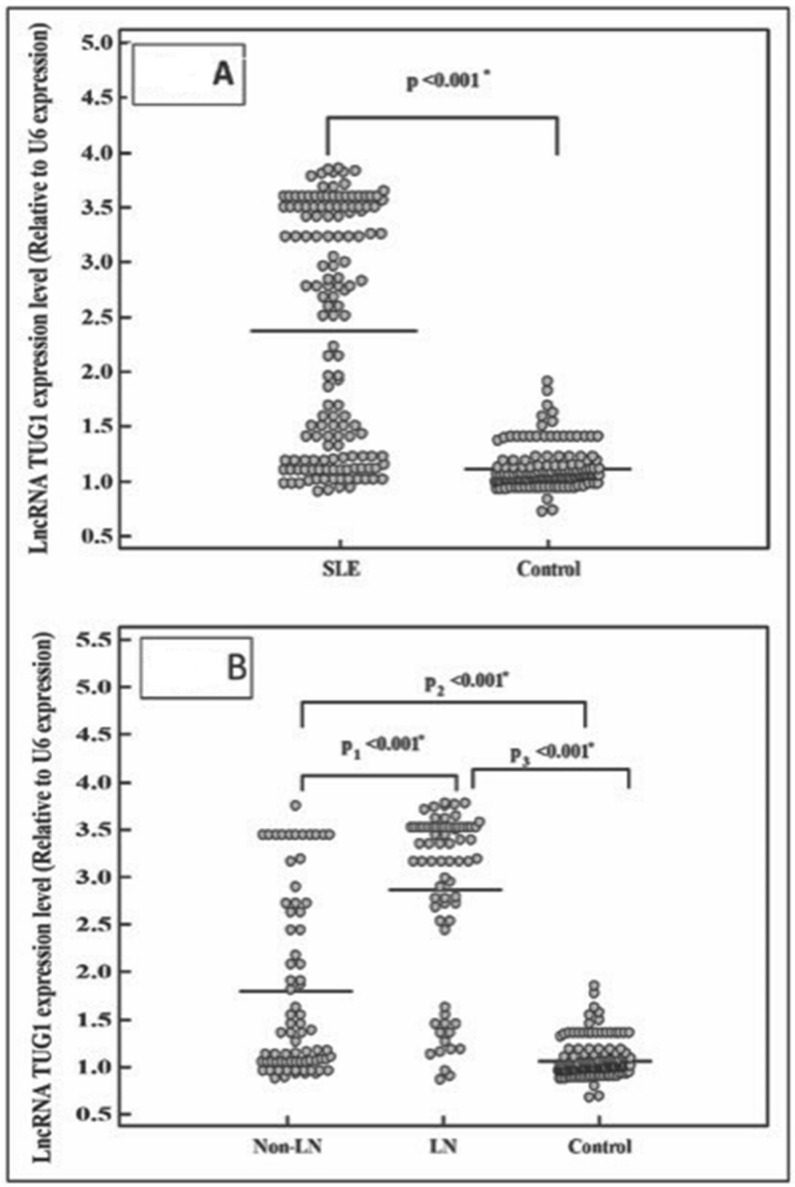
LncRNA TUG1 expression level in the studied groups. (**A**): TUG1 level between SLE cases and healthy control, (**B**): TUG1 level between LN and non-LN. *: Statistically significant at *p* ≤ 0.05.

In the current study, TUG 1 expression level was measured by qRT-PCR, and there was a significantly higher level of TUG 1 in SLE cases compared to healthy control (*p* < 0.001). Additionally, within SLE cases, the TUG 1 expression in LN group had a significantly higher level than in non-LN cases (*p* < 0.001), Figure 1 and Figure 2.

### 2.7. Relation of TUG 1 Gene Polymorphism and TUG 1 Level

**Figure 3:**
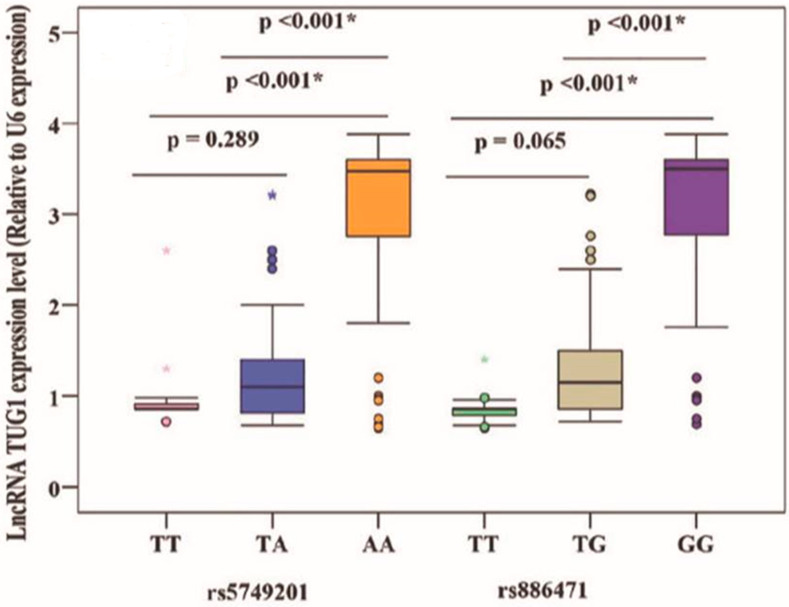
Relation of TUG 1 gene polymorphism and TUG 1 level. *: Statistically significant at *p* ≤ 0.05.

It was found that AA rs5749201 and GG rs886471 were associated with higher levels of TUG 1 than other genotypes (*p* < 0.001), Figure 3.

### 2.8. Predictors of Lupus Nephritis

Using univariate regression analysis, it was found that discoid rash (*p* = 0.002), a very high SLEDAI score (*p* = 0.001), and AA rs5749201 (*p* = 0.034) could significantly predict the incidence of LN in SLE cases (Table 7).

## 3. Discussion

SLE is an autoimmune inflammatory disorder, and patients with SLE are more likely to be associated with complications, such as LN, cardiac disorders, and infections [1]. Although the current therapy improves and can control the disease, comorbidity and death persist, indicating that additional medical approaches are needed. Thus, a better understanding of the molecular pathogenesis of the disease for earlier diagnosis and intervention is a critical issue.

There is a growing understanding of the role of lncRNA in disease pathogenesis by controlling transcriptional and post-transcriptional genes [20].

The association of lncRNA TUG1 (rs5749201 and rs886471) has not been studied before, and to the best of our knowledge, this is the first study investigating this association and evaluating its relationship with LN. Our results demonstrated that TUG1 rs5749201 and rs886471 gene polymorphisms were associated with an increase in the risk of SLE and significantly increased the susceptibility of LN where the AA, AT, and A alleles of rs5749201 and the GG, GT, and G alleles of rs886471, in addition to the dominant of both SNPs, had a higher risk of SLE. However, the recessive genotype has no risk. Only homozygous genotypes, alleles, and dominant and recessive models increased LN risk. In this context, only two recent studies have reported a link between TUG1 gene polymorphism and other diseases. One study was conducted by Duan et al., who reported that TUG1 gene polymorphism increased the risk of osteoarthritis [16], and the second study by Mohammad et al., who stated that the association of TUG1 gene polymorphism increased the risk of diabetic retinopathy [21], which indicated that TUG1 variants might contribute to inflammatory disorders.

A previous study linked TUG1 with inflammation as TUG1 knockdown decreased IL-6 and TNF in an animal model of atherosclerosis as well as upregulated inflammatory factor expression by sponging miR-133a in ox-LDL-treated macrophages [22].

To assess the historical demography and gene flow resulting from allele hybridization, the present study evaluated the haplotype frequency in patients and controls. It was revealed that the AG haplotype increased the risk of SLE and LN.

The current study demonstrated that the AA rs5749201 genotype was associated with delayed disease onset. A previous study reported that childhood-onset diseases are more severe than adult-onset diseases and that patients with later-onset diseases have more cardiopulmonary complications than those with adult-onset diseases [23,24]. This study demonstrated that the homozygous genotype of both SNPs is significantly more prevalent in males, which is consistent with a recent study that reported that SLE is rarer in men than in women and that men have a more severe disease phenotype [25].

Moreover, the results reflected that TUG1 gene polymorphism was associated with prolonged disease duration and unfavorable clinical laboratory parameters, such as more arthritis, renal affection, leucopenia and thrombocytopenia, higher CRP, ESR, C3, and C4 levels, and a higher percentage of dsDNA. Disease activity is an important factor in the prognosis of a disease. In the present study, there was an association between the TUG1 homozygous genotype and a higher SLEDAI score, indicating that TUG1 gene polymorphism affects the clinical and laboratory phenotype of the disease, with more severe illness in homozygous genotypes, and that TUG1 may be involved in the pathogenesis and prognosis of SLE.

We chose these TUG1 SNPs based on bioinformatic analysis, which showed that the SNP loci may affect the transcriptional level of TUG1 as rs5749201 T > A is located 3 upstream of the gene and rs886471 T > G is an upstream transcript variant that could affect the transcriptional level of TUG1.

Interestingly, in the current study, we found that the TUG1 level was significantly higher in total SLE patients than in the control and in LN patients than in non-LN ones. Additionally, the GG and AA genotypes of both SNPs were associated with significantly higher levels of TUG1 than the other genotypes. This may indicate that the TUG1 gene polymorphism is associated with SLE and LN because of the regulation of TUG1 gene expression and function; however, more experimental model studies are required to investigate the signaling pathway of action of TUG1. Few recent studies indicated the signaling pathway of TUG1 by increasing TNF via the activation of the NF-KB pathway by regulating miR-26a/HMGB1 [26] and development of atherosclerosis through modulating miR-21/phosphatase [8].

TUG1 was discovered to be involved in neuroinflammation by sponging miR 145a-5b, and TUG1 silencing altered the microglia phenotype (from M1 to M2) by downregulation pro-inflammatory cytokines and upregulating anti-inflammatory cytokines [27].

Moreover, using univariate and multivariate analysis, the power of TUG1 AA rs5749201 with very high SLEDAI score and the presence of discoid rash are the only predictors of LN. The limitations of this study are the lack of a mechanism for understanding the role of TUG1 gene polymorphism in renal pathology and the lack of patient follow-up to determine whether TUG1 gene polymorphism has an impact on the treatment of the diseases. These limitations are recommended for further research. Moreover, the cost of genetic testing in comparison with other investigations should be considered in further studies.

In conclusion, our findings demonstrated that TUG1 gene polymorphism and TUG1 level are associated with an increased risk of SLE and LN in the Egyptian population, and the homozygous genotype is correlated with a more progressive disease. Therefore, the TUG1 variant and TUG1 level can be used as molecular biomarkers for the risk of SLE and LN and prognostic factors for the disease. This is the first study to report the association between TUG1 polymorphism and its expression level with SLE and LN. Additional studies with a larger sample size and different ethnic populations are needed to confirm our results, with a focus focusing on the pathophysiology of renal pathology.

## 4. Materials and Methods

The present study was conducted at Clinical Pathology, internal medicine, physical Medicine, Rheumatology and Rehabilitation Departments, College of Medicine-Menoufia University.

### 4.1. Study Design and Patient Groups

This study was conducted on 145 patients diagnosed with SLE in addition to 145 age- and gender -matched volunteers as control.

Inclusion criteria: All enrolled patients fulfilled the diagnostic criteria for SLE defined by the Systemic Lupus International Collaborating Clinics criteria of 2012. Written consent was obtained from all subjects. The study was conducted according to the guidelines of the Declaration of Helsinki 1964, and approved by the Ethics Committee of the Faculty of medicine, Menoufia University.

Exclusion criteria: Patients with autoimmune diseases other than SLE, extreme age group, and drug-induced lupus were excluded from the study.

### 4.2. Clinical Assessment

History and Clinical datawere taken from all patients; including age, onset and duration of disease, photosensitivity, malar rash, falling of hair, discoid lesion, arthritis, oral ulcer, neurological manifestations, clinical signs of lupus nephritis (LN), and secondary antiphospholipid syndrome were collected from patients’ records.

Disease activity was determined for each patient using the modified Systemic Lupus Erythematosus Disease Activity Index (SLEDAI): SLEDAI = 0 (no activity), SLEDAI = 1–5 (mild activity), SLEDAI = 6–10 (moderate activity), SLEDAI = 11–19 (high activity), SLEDAI ≥ 20 (very high activity).

### 4.3. Laboratory Evaluation

#### 4.3.1. Routine Laboratory and Autoimmune Panel

Complete blood count (APX pentra XL80), kidney function tests, creatinine/protein ratio (Beckman, Brea, CA, USA), complement C3 and C4 level (immunoturbidimetric assay (Orion Diagnostica Turbox, New York, NY, USA), antinuclear antibodies (ANA), ANA pattern (Immuneflurescence microscope), anti-double stranded DNA (dsDNA) antibodies (Immuneflurescence microscope) and anticardiolipin antibodies (ELISA, Demeditec Diagnostics GmbH, Hamburg, Germany) were all done for each patient.

#### 4.3.2. Quantitative Real-Time Quantitative PCR (qRT-PCR) for lncRNA TUG1 Expression Level

##### RNA Extraction from Plasma and Reverse Transcription

4 mL peripheral blood was collected from the subjects and centrifuged at 3000× *g* for 10 min and the supernatant was collected. Total RNA was isolated from plasma using miRNeasy Mini Kit (Qiagen, Germantown, MD, USA) according to the manufacturer’s instructions. RNA levels were assessed using system Nanophotometer N60 IMPLEN GNBH (Munich, Germany). To prepare for the RT phase, the extract was kept at −80 °C. For reverse transcription and cDNA synthesis, we employed the Revert Aid kit (Thermo Scientific, Waltham, MA, USA). The following reactions were carried out on ice in a total volume of 20 µL: To make a total volume of 12 µL, 10 µL RNA was added to 1 µL of random hexamer and 1 µL of nuclease-free water. This mixture was then incubated at 64 °C for 5 min before being chilled on ice. Second, we added a total of 20 µL to the previously described mixture by adding 4 µL of 5× reaction buffer, 1 µL of RiboLock RNase inhibitor, 2 µL of 10 mM dNTPs, and 1 µL of Revert Aid RT. The thermal cycler Biometra T professional thermocycler 070-851 (Darmstadt, Germany) was used for one cycle of incubation, with the following temperatures: 25 °C for 5 min, 42 °C for 60 min, and termination at 70 °C for 4 min. Prior to real-time PCR, the generated cDNA was stored at −20 °C.

##### Quantification of LncRNA (TUG1) Expression by Real-Time PCR Technique

qRT-PCR was performed using the synthesized cDNA as a template to detect the level of lncRNA TUG1. U6 was used as an internal control and the kit was Maxima SYBR green qPCR Master mix (Thermo scientific, USA). The primer sequence of lncRNA TUG1 is F: 5′-TAG CAG TTC CCC AAT CCT TG-3′ and R: 5′-CAC AAA TTC CCA TCA TCC C-3′. The primer sequence of U6 is F: 5′-CTC GCT TCG GCA GCA CA-3′ and R: 5′-AAC GCT TCA CGA ATT TGC GT-3′. PCR reaction conditions: 40 cycles of 95 °C for 30 s, 95 °C for 5 s, and 60 °C for 34 s. Final fluorescence detection and data analysis were performed using a 7500 ABI PRISM system v.2.0.1. (Applied Biosystems, Foster City, CA, USA). The relative quantitation values of the expression levels were measured in relation to mean value of expression in control sample using the 2^−(ΔΔ CT)^.

#### 4.3.3. DNA Extraction and Detection of Genotyping

DNA was extracted using QIAamp DSP DNA Blood Mini Kit (QIAGEN, USA) and was stored in a refrigerator at −20 °C. The PCR amplification was performed using specific primers (Macrogen, Seoul, South Korea). The primer sequences are as follows: TUG1 rs5749201 F: 5′-TGC CTG CAT TTA CTG TCT TTG C-3′, R: 5′-TGT TGT GGT GTA TGT GGG CA-3′; TUG1 rs886471 F: 5′-ATG TCT AGG CTG TGT GGT TGG-3′, R: 5′-GAG CCC GCT TGC TAA AAG TC-3′, with a total volume of 25 μL including the following: 12.5 µL of a ready to use master mix (Promega, Woods Hollow Road Madison, WI, USA), 1.5 µL of each primer (0.5 µmol), 5.5 µL of template DNA (100–500 ng) and 1.0 µL of sterile deionized water. The thermal cycler profile (Biometra T professional thermocycler 070-851, Germany)). The cycling conditions included an initial denaturation step at 95 °C for 5 min followed by 35 cycles at 95 °C for 30 s, 60 °C for 30 s, 72 °C for 30 s and a final extension of 7 min at 72 °C. After PCR amplification, the products were purified and then subjected to Sanger sequencing. By comparing the sequencing results to the sequencing data in the dbSNP database, the genotypes of TUG1 rs5749201 and rs886471 were determined.

### 4.4. Statistical Analysis

Data were fed to the computer and analyzed using IBM SPSS software package version 20.0. (Armonk, NY, USA: IBM Corp). Categorical data were represented as numbers and percentages. The differences between the variables were assessed by chisquare test, Fisher Exact and Monte Carlo correction, Mann Whitney or Kruskal–Wallis one-way analysis. Quantitative data were expressed as range (minimum and maximum), mean, standard deviation and median. Student t-test was used to compare two groups while one way ANOVA test was used for comparing the three studied groups and followed by Post Hoc test. Odd ratio was used to calculate the ratio of the odds and 95% Confidence Interval of an event. Logistic regression analysis was used to detect the most independent factor for affecting Active AS. Significance of the obtained results was judged at the 5% level.

## Figures and Tables

**Table 1:** Distribution of the studied cases according to different parameters in SLE group (*n* = 145).

	No. (%)
**Age (years)**	40.3 ± 7.77
**Sex**	
Male	21 (14.5%)
Female	124 (85.5%)
**Disease duration (years)**	6.26 ± 5.63
**Malar rash**	81 (27.9%)
**Discoid rash**	38 (13.1%)
**Photosensitivity**	22 (7.6%)
**Arthritis**	104 (35.9%)
**Mucosal ulcers**	41 (14.1%)
**Renal affection**	70 (24.1%)
**Corticosteroid**	145 (50%)
**Immunosuppressive**	72 (24.8%)
**Anemic (Hb < 12.5)**	130 (89.7%)
**WBC Leukopenia**	103 (71.0%)
**Platelet count Thrombocytopenia**	89 (61.4%)
**Albumin in urine**	
Normal	88 (60.7%)
Micro albuminuria	29 (20.0%)
Macro albuminuria	28 (19.3%)
**Protein Creatinine ratio**	0.63 ± 0.83
**CRP**	51.5 ± 21.4
**ESR**	55.1 ± 31.1
**COOMB,S**	58 (40%)
**Serum Creatinine (mg/dL)**	1.40 ± 0.64
**Serum urea**	62 ± 37.3
**Anti-ds-DNA**	124 (85.5%)
**ANA**	140 (96.6%)
**C3 (mg/dL)**	81.9 ± 33.7
**C4 (mg/dL)**	14.5 ± 8.79
**SLEDAI**	11.08 ± 6.64
Mild (1–5)	41 (28.3%)
Moderate (6–10)	30 (20.7%)
High (11–19)	47 (32.4%)
Very high (≥20)	27 (18.6%)
**LN**	70 (48.3%)

Quantitative data was expressed using Mean ± SD. SD: Standard deviation.

**Table 2:** Hardy-Weinberg for genotype frequencies.

	SLE (*n* = 145)	LN (*n* = 70)	Non-LN (*n* = 75)	Control (*n* = 145)
**rs5749201**				
**^HW^χ^2^ (*p*)**	3.629 (0.057)	3.926 (0.048)	0.260 (0.610)	3.542 (0.060)
**rs886471**				
**^HW^χ^2^ (*p*)**	3.256 (0.071)	1.643 (0.200)	0.059 (0.807	3.734 (0.053)

**^HW^χ^2^:** Chi square for goodness of fit for Hardy-Weinberg equilibrium (If *p* < 0.05—not consistent with HWE.).

**Table 3:** Comparison between the two studied groups according to rs5749201 and rs886471.genotype frequencies.

	SLE (*n* = 145)	Control^®^ (*n* = 145)	χ^2^ (*p*)	OR (LL–UL 95%C.I)	*p*_0_
**rs5749201**					
**Genotype**					
TT^®^	15 (10.3%)	(60 + 0) 60 (41.4%)	55.696 * (<0.001 *)	1.0	
TA	48 (33.1%)	(19 + 39) 58 (40.0%)		3.310 (1.672–6.553)	0.001 *
AA	82 (56.6%)	(66 − 39) 27 (18.6%)		12.148 (5.951–24.799)	<0.001 *
**Dominant** TA + AA vs. TT^®^	130/15	85/60	36.419 * (<0.001 *)	6.118 (3.263–11.468)	<0.001 *
**Recessive** AA vs. TT + TA^®^	82/63	27/118	44.465 * (<0.001 *)	5.688 (3.343–9.680)	<0.001 *
**Allele**	**(*n* = 290)**	**(*n* = 290)**			
T^®^	78 (26.9%)	178 (61.4%)	69.927 * (<0.001 *)	1.0	
A	212 (73.1%)	112 (38.6%)		4.320 (3.041–6.136)	<0.001 *
**rs886471**					
**Genotype**					
TT^®^	16 (11.0%)	(65 + 0) 65 (44.8%)	59.351 (<0.001 *)	1.0	
TG	50 (34.5%)	(17 + 39) 56 (38.6%)		3.627 (1.862–7.066)	<0.001 *
GG	79 (54.5%)	(63 − 39) 24 (16.6%)		13.372 (6.557–27.272)	<0.001 *
**Dominant** TG + GG vs. TT^®^	129/16	80/65	41.130 * (<0.001 *)	6.551 (3.545–12.105)	<0.001 *
**Recessive** GG vs. TT + TG^®^	79/66	24/121	45.545 * (<0.001 *)	6.035 (3.545–12.105)	<0.001 *
**Allele**	**(*n* = 290)**	**(*n* = 290)**			
T^®^	82 (28.3%)	186 (64.1%)	75.025 * (<0.001 *)	1.0	
G	208 (71.7%)	104 (35.9%)		4.537 (3.195–6.441)	<0.001 *
**Haplotype**	**(*n* = 290)**	**(*n* = 290)**			
TT^®^	73 (25.2%)	178 (61.4%)	82.464 * (^MC^ *p* < 0.001 *)	1.0	
TG	5 (1.7%)	0 (0.0%)		–	0.999
AT	9 (3.1%)	8 (2.8%)		2.743 (1.019–7.387)	0.046 *
AG	203 (70.0%)	104 (35.9%)		4.759 (3.318–6.826)	<0.001 *

^®^: Reference group; OR: Odd’s ratio; C.I: Confidence interval; LL: Lower limit; UL: Upper Limit; χ^2^: Chi square test; MC: Monte Carlo; *p*_0_: *p* value for binary logistic regression; *p*: *p* value for comparing between the two studied groups; *: Statistically significant at *p* ≤ 0.05.

**Table 4:** Comparison between the two studied subgroups according to rs5749201 and rs886471.

Genotype	LN (*n* = 70)	Non-LN^®^ (*n* = 75)	χ^2^ (*p*)	*p*_0_	OR (LL–UL 95% C.I)
**rs5749201**					
**Genotype**					
TT^®^	2 (2.9%)	13 (17.3%)	42.500 * (<0.001)		1.0
TA	9 (12.9%)	39 (52.0%)		0.631	1.50 (0.286–7.856)
AA	59 (84.3%)	23 (30.7%)		<0.001 *	16.67 (3.487–79.72)
**Dominant** TA + AA vs. TT^®^	68/2	62/13	8.181 * (0.004)	0.012 *	7.129 (1.547–32.86)
**Recessive** AA vs. TT + TA^®^	59/11	23/52	42.365 * (<0.001)	<0.001 *	12.13 (5.397–27.25)
**Allele**	**(*n* = 140)**	**(*n* = 150)**			
T^®^	13 (9.3%)	65 (43.3%)	42.693 * (<0.001)		1.0
A	127 (90.7%)	85 (56.7%)		<0.001 *	7.471 (3.878–14.393)
**rs886471**					
**Genotype**					
TT^®^	2 (2.9%)	14 (18.7%)	36.175 * (<0.001)		1.0
TG	12 (17.1%)	38 (50.7%)		0.336	2.211 (0.439–11.142)
GG	56 (80.0%)	23 (30.7%)		<0.001 *	17.04 (3.585–81.03)
**Dominant** TG + GG vs. TT ^®^	68/2	61/14	9.218 * (0.002)	0.008 *	7.803 (1.704–35.73)
**Recessive** GG vs. TT + TG^®^	56/14	23/52	35.533 * (<0.001)	<0.001 *	9.043 (4.212–19.42)
**Allele**	**(*n* = 140)**	**(*n* = 150)**			
T^®^	16 (11.4%)	66 (44.0%)	37.880 * (<0.001)		1.0
G	124 (88.6%)	84 (56.0%)		<0.001 *	6.089 (3.301–11.234)
**Haplotype**	**(*n* = 140)**	**(*n* = 150)**			
TT^®^	12 (8.6%)	61 (40.7%)	45.972 * (^MC^ *p* < 0.001)		1.0
TG	1 (0.7%)	4 (2.7%)		0.837	1.271 (0.130–12.388)
AT	4 (2.9%)	5 (3.3%)		0.058	4.067 (0.951–17.392)
AG	123 (87.9%)	80 (53.3%)		<0.001 *	7.816 (3.960–15.426)

^®^: Reference group; OR: Odd’s ratio; C.I: Confidence interval; LL: Lower limit; UL: Upper Limit; χ^2^: Chi square test; MC: Monte Carlo; *p*_0_: *p* value for binary logistic regression; *p*: *p* value for comparing between the two studied groups; *: Statistically significant at *p* ≤ 0.05.

**Table 5:** Relation between rs5749201 and different parameters in SLE patients (*n* = 145).

	rs5749201			Test of Sig.	*p*	TT vs. TA	TT vs. AA	TA vs. AA
	TT (*n* = 15)	TA (*n* = 48)	AA (*n* = 82)					
**Age (years)**	36.1 ± 5.95	37.4 ± 8.89	42.8 ± 6.37	F = 11.382 *	<0.001 *	0.837	0.004 *	<0.001 *
**Sex**								
Male	0 (0%)	3 (6.3%)	18 (22%)	χ^2^ = 8.860 *	0.012 *	1.000 ^a^	0.066 ^a^	0.019 *
Female	15 (100%)	45 (93.8%)	64 (78%)					
**Disease Duration (years)**	2.80 ± 1.07	4.09 ± 3.19	8.15 ± 6.45	H = 16.053 *	<0.001 *	0.466	0.003 *	0.001 *
**Malar rash**	10 (66.7%)	21 (43.8%)	50 (61.0%)	χ^2^ = 4.436	0.109	–	–	–
**Discoid rash**	3 (20.0%)	17 (35.4%)	18 (22.0%)	χ^2^ = 3.172	0.205	–	–	–
**Photosensitivity**	3 (20.0%)	8 (16.7%)	11 (13.4%)	χ^2^ = 0.552	0.759	–	–	–
**Arthritis**	4 (26.7%)	42 (87.5%)	58 (70.7%)	χ^2^ = 20.95 *	<0.001 *	<0.001 *^a^	0.001 *	0.029 *
**Mucosal ulcers**	7 (46.7%)	8 (16.7%)	26 (31.7%)	χ^2^ = 6.167 *	0.046 *	0.033 *^a^	0.261	0.060
**Renal affection**	2 (13.3%)	9 (18.8%)	59 (72.0%)	χ^2^ = 42.5 *	<0.001 *	1.0 ^a^	<0.001 *	<0.001 *
**Anemia**	14 (93.3%)	39 (81.3%)	77 (93.9%)	χ^2^ = 4.935	0.067 ^b^	–	–	–
**WBC Leukopenia**	15 (100%)	22 (45.8%)	66 (80.5%)	χ^2^ = 24.494 *	<0.001 *	<0.001 *	0.069 ^a^	<0.001 *
**thrombocytopenia**	1 (6.7%)	23 (47.9%)	65 (79.3%)	χ^2^ = 33.682 *	<0.001 *	0.004 *	<0.001 *^a^	<0.001 *
**CRP**	52.3 ± 18.0	45.2 ± 24.9	55.1 ± 19.1	H = 11.579 *	0.003 *	0.587	0.015 *	0.004 *
**ESR**	35.0 ± 4.14	42.3 ± 30.8	66.2 ± 29.5	H = 26.877 *	<0.001 *	0.221	<0.001 *	<0.001 *
**Protein/Creatinine ratio**	0.22 ±0.19	0.42 ± 0.64	0.83 ± 0.93	H = 31.173 *	<0.001 *	0.153	0.038 *	<0.001 *
**C3 (mg/dL)**	105 ±17.7	90.5 ± 34.9	72.6 ± 32.0	H = 22.064 *	<0.001 *	0.096	<0.001 *	0.001 *
**C4 (mg/dL)**	22.3 ± 2.84	16.5 ± 8.10	11.9 ± 8.81	H = 29.120 *	<0.001 *	0.034 *	<0.001 *	<0.001 *
**ANA**	15 (100%)	44 (91.7%)	81 (98.8%)	χ^2^ = 4.002	0.090 ^b^	–	–	–
**ds-DNA**	3 (20.0%)	42 (87.5%)	79 (96.3%)	χ^2^ = 59.90 *	<0.001 *	<0.001 *^a^	<0.001 *^a^	0.075 ^a^
**SLEDAI**								
High (11–19)	0 (0.0%)	7 (14.6%)	40 (48.8%)	χ^2^ = 24.186 *	<0.001 *	0.182 ^a^	<0.001 *	<0.001 *
Very high (≥20)	3 (20.0%)	2 (4.2%)	22 (26.8%)	χ^2^ = 10.283 *	0.006 *	0.083 ^a^	0.753 ^a^	0.001 *

Quantitative data was expressed using Mean ± SD; SD: Standard deviation; χ^2^: Chi square test; ^a^: Fisher Exact; ^b^: Monte Carlo; F: F for One way ANOVA test, Pairwise comparison bet. each 2 groups was done using Post Hoc Test (Tukey); H: H for Kruskal Wallis test, Pairwise comparison bet. each 2 groups was done using Post Hoc Test (Dunn’s for multiple comparisons test). *: Statistically significant at *p* ≤ 0.05.

**Table 6:** Relation between rs886471 and different parameters in SLE patients (*n* = 145).

	rs886471			Test of Sig.	*p*	TT vs. TG	TT vs. GG	TG vs. GG
	TT (*n* = 16)	TG (*n* = 50)	GG (*n* = 79)					
**Age (years)**	37.6 ± 5.1	37.2 ± 9.14	42.9 ± 6.27	F = 10.725 *	<0.001 *	0.980	0.023 *	<0.001 *
**Sex**								
Male	0 (0%)	4 (8.0%)	17 (21.5%)	χ^2^ = 7.564 *	0.023 *	0.565	0.067 ^a^	0.043 *
Female	16 (100%)	46 (92.0%)	62 (78.5%)					
**Disease duration (years)**	4.03 ± 3.23	3.73 ± 3.01	8.30 ± 6.43	H = 18.365 *	<0.001 *	0.589	0.031 *	<0.001 *
**Malar rash**	10 (62.5%)	22 (44.0%)	49 (62.0%)	χ^2^ = 4.356	0.113	–	–	–
**Discoid rash**	3 (18.8%)	17 (34.0%)	18 (22.8%)	χ^2^ = 2.509	0.285	–	–	–
**Photosensitivity**	5 (31.3%)	7 (14.0%)	10 (12.7%)	χ^2^ = 3.655	0.161	–	–	–
**Arthritis**	4 (25.0%)	43 (86.0%)	57 (72.2%)	χ^2^ = 22.25 *	<0.001 *	<0.001 *^a^	<0.001 *	0.066
**Mucosal ulcers**	8 (50.0%)	7 (14.0%)	26 (32.9%)	χ^2^ = 9.585 *	0.008 *	0.006 *^a^	0.194	0.016 *
**Renal affection**	2 (12.5%)	12 (24.0%)	56 (70.9%)	χ^2^ = 36.2 *	<0.001 *	0.488 ^a^	<0.001 *	<0.001 *
**Anemia**	16 (100%)	40 (80%)	74 (93.7%)	χ^2^ = 8.245 *	0.016 *	0.102 ^a^	0.585 ^a^	0.018 *
**Leucopenia**	15 (93.8%)	24 (48.0%)	64 (81.0%)	χ^2^ = 20.729 *	<0.001 *	0.001 *	0.293 ^a^	<0.001 *
**thrombocytopenia**	1 (6.3%)	23 (46.0%)	65 (82.3%)	χ^2^ = 40.059 *	<0.001 *	0.004 *	<0.001 *^a^	<0.001 *
**CRP**	46.2 ± 23.1	44.7 ± 24.9	56.9 ± 17.0	H = 18.910 *	<0.001 *	0.357	0.001 *	<0.001 *
**ESR**	44.4 ± 29.6	39.2 ± 26.7	67.2 ± 29.0	H = 27.795 *	<0.001 *	0.960	0.001 *	<0.001 *
**Protein/creatinine ratio**	0.43 ± 0.67	0.43 ± 0.64	0.80 ± 0.93	H = 28.119 *	<0.001 *	0.112	0.069	<0.001 *
**C3 (mg/dL)**	103 ± 20.4	92.5 ± 33.9	70.9 ± 31.7	H = 24.682 *	<0.001 *	0.238	<0.001 *	<0.001 *
**C4 (mg/dL)**	21.6 ± 3.76	17.2 ± 8.12	11.4 ± 8.56	H = 35.036 *	<0.001 *	0.084	<0.001 *	<0.001 *
**ANA**	16 (100%)	46 (92.0%)	78 (98.7%)	χ^2^ = 3.671	0.105 ^b^	–	–	–
**ds-DNA**	5 (31.3%)	43 (86.0%)	76 (96.2%)	χ^2^ = 45.34 *	<0.001 *	<0.001 *^a^	<0.001 *^a^	0.046 *^a^
**SLEDAI**								
High (11–19)	0 (0%)	7 (14.0%)	40 (50.6%)	χ^2^ = 27.382 *	<0.001 *	0.181 ^a^	<0.001 *	<0.001 *
Very high (≥20)	2 (12.5%)	4 (8.0%)	21 (26.6%)	χ^2^ = 7.422 *	0.024 *	0.627 ^a^	0.342 ^a^	0.009 *

Quantitative data was expressed using Mean ± SD; SD: Standard deviation; χ^2^: Chi square test; ^a^: Fisher Exact; ^b^: Monte Carlo; F: F for One way ANOVA test, Pairwise comparison bet. each 2 groups were done using Post Hoc Test (Tukey); H: H for Kruskal Wallis test, Pairwise comparison bet. each 2 groups were done using Post Hoc Test (Dunn’s for multiple comparisons test); *p*: *p* value for relation between rs5749201 and different parameters; *: Statistically significant at *p* ≤ 0.05.

**Table 7:** Univariate and multivariate logistic regression analysis for the parameters affecting LN (*n* = 70 vs. 75) for SLE groups.

	^#^ Multivariate		^#^ Univariate	
	OR (95% C.I = LL–UL)	*p*	OR (95% C.I = LL–UL)	*p*
**Disease duration**	0.990 (0.934–1.049)	0.725		
**Presence of Discoid rash**	0.331 (0.149–0.736)	0.007 *	0.133 (0.037–0.476)	0.002 *
**Presence of Arthritis**	0.488 (0.233–1.021)	0.057		
**Presence of Mucosal ulcer**	1.351 (0.654–2.789)	0.416		
**High SLEDAI (11–19)**	1.727 (0.855–3.486)	0.128		
**Very high SLEDAI (≥20)**	6.417 (2.272–18.121)	<0.001 *	13.861 (3.093–62.113)	0.001 *
**rs5749201 genotypes**				
**TT^®^**	1.000		1.000	
**TA**	1.500 (0.286–7.856)	0.631	0.907 (0.095–8.673)	0.932
**AA**	16.674 (3.487–79.724)	<0.001 *	70.794 (1.369–3662.20)	0.034 *
**rs886471 genotypes**				
**TT^®^**	1.000		1.000	
**TG**	2.211 (0.439–11.142)	0.336	6.262 (0.448–87.503)	0.173
**GG**	17.043 (3.585–81.032)	<0.001 *	0.235 (0.008–7.130)	0.406
**Lnc RNA TUG1**	2.639 (1.887–3.689)	<0.001 *	1.701 (0.969–2.984)	0.064

OR: Odd’s ratio; C.I: Confidence interval; LL: Lower limit; UL: Upper Limit; ^#^: All variables with *p* < 0.05 was included in the multivariate; *: Statistically significant at *p* ≤ 0.05.

## Data Availability

The datasets generated during and/or analyzed during the current study are available from the corresponding author on reasonable request.
